# Poisoning Pickles

**Published:** 1875-11

**Authors:** 


### POISONING PICKLES.

The *American Grocer* alludes to the beutifying processes by which pickles are rendered poisonous. There is, in the making of pickles, a feature of serious importance, respecting which every member of the community should be apprised. We allude to the artificial coloring to give, as is supposed, a more inviting appearance to pickles. The best and freshest pickles lose some of their color in the salting process, which, to make them presentable when in bottles and so satisfy an imperative demand of the trade, it is a part of the manufacturer's business to "restore," although the term is falsely applied. To *add* this green color, in the last stage of the freshening process, the top of the vat of pickles, heated to boiling point, is covered for a time with a sheet of clean copper, and after this the pickles come out apparently as fresh a green as when gathered. It is claimed that this process is no more injurious to health than heating water for drinking purposes in any copper vessel, and it is probable that from the abuse of the means the real danger is incurred; nevertheless, we consider the process unnecessary, and one that should be discountenanced. Complaint is made by persons engaged in the regular trade that large quantities of stale, inferior cucumbers, roughly counted, and low in price, are supplied to irresponsible manufacturers, who are thereby enabled to cut prices and greatly hinder legitimate business. To give to such pickles anything like an appearance of freshness, more violent and very reprehensible means are resorted to. In the freshening process the water is heated to a temperature of about 150 degrees Fahrenheit, and bluestone or sulphate of copper is added, which is a deadly poison, and is largely used in sowing wheat to kill the enemies of the seed. This, by heating, impregnates the pickles, coloring the outside a beautiful green, and remaining in the vegetable after it is taken out of the water, put in vinegar, and packed in barrels, kegs, or bottles, for use. So strong is the poison with which pickles are impregnated to produce this green tint, that sometimes its astringency will draw up the mouth when they are bitten, and so dangerous that we heard an old pickle-manufacturer say emphatically that he would not eat a piece of green pickle the size of one of his fingers for ten thousand dollars! We may mention that some time ago a case of wholesale poisoning occurred in New York, which was clearly traced to the eating of these cucumbers; and how many cases of sickness, without apparent cause, may not be owing to the practice? Sometimes, instead of adding bluestone, the outrageous practice of putting into the heated water old sheets of copper from ships' bottoms, corroded with vinegar, is resorted to. When it is remembered ships are sheathed with this expensive article to *kill* the marine animals that encrust the submerged portion of the hull, the deadly effect of such a process may be readly imagined. The knowledge of such facts as these should make purchasers careful as to what pickles they buy, and lead them to deal only with responsible houses. In some countries the sale of green pickles is strictly prohibited. We might mention France as an instance, and a similar law should be enforced in this country. There are some manufacturers, we are glad to say, who have the physical well being of the community at heart, and eschew poison. As a simple rule, the brown-colored pickles are the most wholesome, while they can be, and are packed quite tastefully enough for all practical purposes.

To claim that to dye pickles by the aid of copper in any form, is not dangerous, is a fallacy. Pure water is not impaired by being boiled in copper; but water containing chloride of sodium or acetic acid, as pickle-water does, dissolves the copper and poisons the mixture. Beware of bright green pickles.

